# Beyond pathogenicity: applications of the type III secretion system (T3SS) of *Pseudomonas aeruginosa*

**DOI:** 10.3389/fmicb.2025.1663945

**Published:** 2025-09-02

**Authors:** Tianqi Su, Lin Zhang, Jie Shen, Danyu Qian, Yulei Guo, Zhenpeng Li

**Affiliations:** School of Laboratory Medicine, Shandong Second Medical University, Weifang, China

**Keywords:** *Pseudomonas aeruginosa*, type III secretion system, therapeutic target, protein delivery system, vaccine

## Abstract

The Gram-negative opportunistic pathogen *Pseudomonas aeruginosa* employs its type III secretion system (T3SS) as a pivotal factor in facilitating the injection of effector proteins into host cells. This process disrupts cellular machinery and immune responses, thereby increasing the pathogen’s survival rates. Recent advancements across multiple disciplines have broadened the scope of T3SS research, extending beyond mechanistic investigations to encompass diverse applications in anti-infective therapies, vaccine development, and protein delivery systems. This comprehensive review analyzes the molecular structure and regulatory mechanisms of T3SS, while also exploring its emerging biomedical applications, which include: (1) the development of antimicrobial agents that target T3SS; (2) T3SS-based vaccines; and (3) T3SS-mediated delivery systems. Furthermore, the review discusses current challenges, particularly focusing on the translational hurdles that hinder clinical application.

## 1 Introduction

*Pseudomonas aeruginosa* (*P. aeruginosa*) is a prevalent Gram-negative opportunistic pathogen responsible for a range of severe infections, including those associated with cystic fibrosis, hospital-acquired pneumonia, burn wound infections, and sepsis (Anantharajah et al., 2016; Thi et al., 2020; Zilberberg et al., 2019). The pathogenicity of *P. aeruginosa* is attributed to various virulence mechanisms, with the type III secretion system (T3SS) playing a crucial role in evading the immune system and adapting to the host (Deng W. et al., 2017). The T3SS functions like a syringe, allowing the transfer of effector proteins into host cells, which disrupts cellular signaling, suppresses immune responses, and promotes bacterial spread (Engel and Balachandran, 2009).

Since the identification of T3SS gene clusters in the 1990s, significant advancements have been made in understanding its structural and functional characteristics (Salmond and Reeves, 1993). Recent developments in cryo-electron microscopy, single-cell sequencing, and artificial intelligence have provided insights into the dynamic assembly of the T3SS, the networks of effector-host interactions, and the regulatory pathways involved (Abby et al., 2016; Butan et al., 2019; Horna and Ruiz, 2021b; Williams McMackin et al., 2019). Concurrently, translational applications have emerged, including T3SS-targeted inhibitors that show significant antibacterial efficacy in animal models (Berube et al., 2017; Kim et al., 2014; Luo et al., 2017; Marsden et al., 2016); T3SS-based vaccines that show protective effects in clinical trials (Das et al., 2020; Fakoor et al., 2020b; Fuentes-Valverde et al., 2022; Meynet et al., 2018); and engineered T3SS delivery systems designed to transport functional proteins (Bichsel et al., 2011; Epaulard et al., 2006; Panthel et al., 2006). Nevertheless, challenges remain, particularly concerning the heterogeneity of *P. aeruginosa*, the complexity of host immune responses, and barriers to clinical translation.

This review aims to summarize the advancements in fundamental research related to the T3SS and its potential applications in the fields of biomedicine and bioengineering. Additionally, it seeks to assess current limitations and propose future directions for T3SS applications.

## 2 T3SS in *Pseudomonas aeruginosa*

**FIGURE 1:**
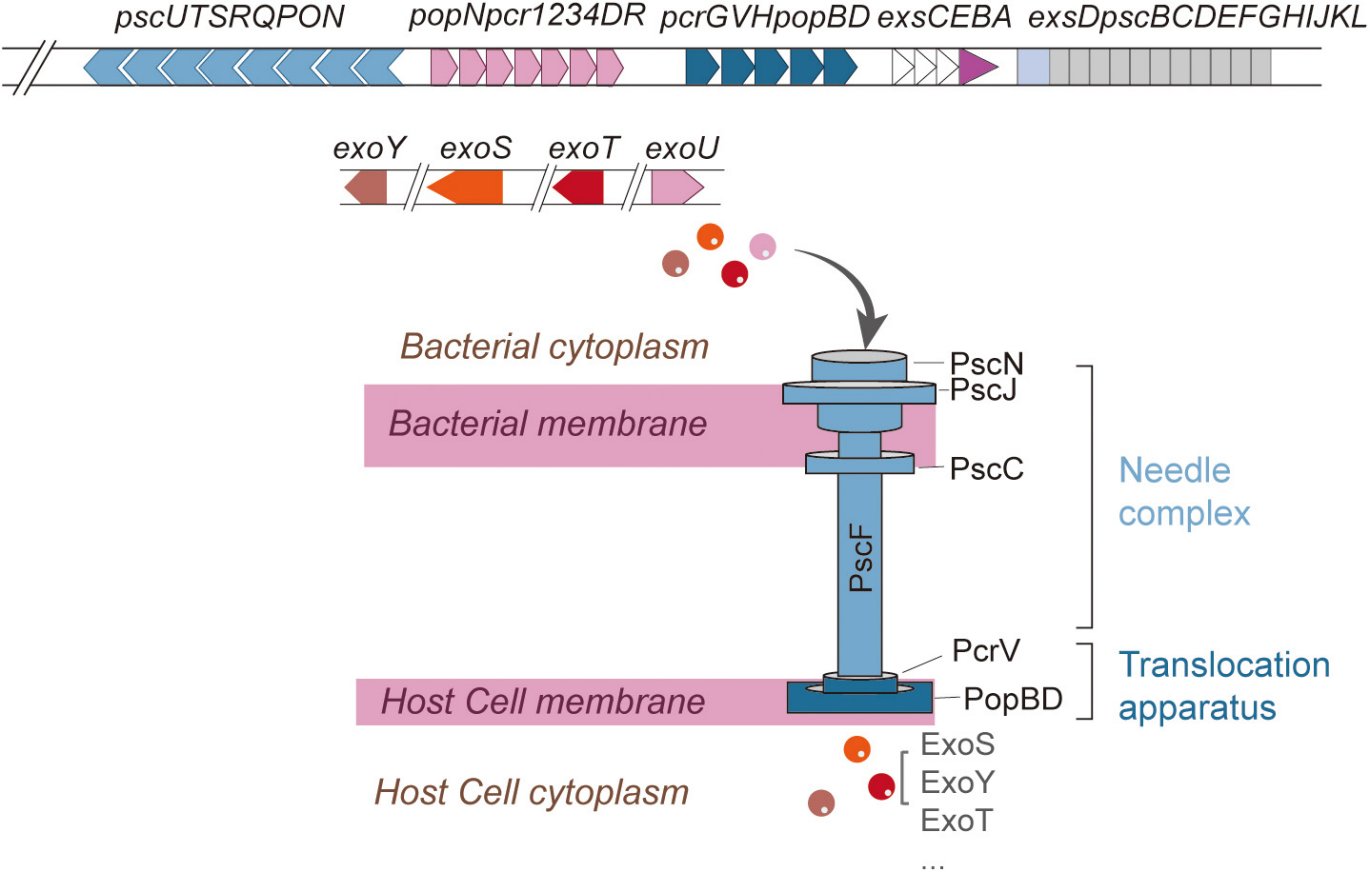
Structure of T3SS in *Pseudomonas aeruginosa*. The five components of T3SS (the needle complex, translocation apparatus, regulatory system, effector proteins, and chaperones) and names of the proteins which constitute each structural component. Structural and regulatory genes are encoded in five consecutive operons, while effectors and chaperones are encoded in other regions of the genome.

Type III secretion system is a multi-subunit protein complex employed by Gram-negative pathogens to initiate and sustain infections (Lara-Tejero and Galán, 2019). T3SS exhibits significant structural conservation across various pathogens, including *Chlamydia trachomatis*, *Escherichia coli*, and *P. aeruginosa* (Hu et al., 2017). Functioning as a biological syringe, the T3SS directly translocates effector proteins into host cells (Pendergrass and May, 2019). In relation to *P. aeruginosa*, the genetic framework of T3SS is organized into five main operons (*pscNOPQRSTU, popNpcr1234DR, pcrGVHpopBD, exsCEBA, exsDpscBCDEFGHIJKL*) (Figure 1), encoding structural and regulatory proteins, while effector proteins (*exoS, exoT, exoU, exoY, pemA, pemB*) and chaperones (*spcS, spcU*) are encoded by additional genomic loci (Barbieri and Sun, 2004; Burstein et al., 2015; Horna and Ruiz, 2021b). Some studies have identified potential novel effector proteins (PemC, PemF) and uncharacterized T3SS-associated proteins (e.g., PscH, Pcr1, Pcr2, PcrR) (Horna and Ruiz, 2021b).

### 2.1 The components and substructures of T3SS

Structurally, the T3SS of *P. aeruginosa* resembles a molecular syringe, comprising five principal components: the needle complex, translocation apparatus, regulatory system, effector proteins, and chaperones (Hauser, 2009). The needle complex can be further subdivided into extracellular appendix, membrane components, and cytoplasmic components (Hauser, 2009; Figure 1).

The extracellular appendix consists of a needle-like structure formed by repeating units of the PscF protein (Hauser, 2009; Pastor et al., 2005). The membrane components span from the inner to the outer bacterial membrane and can be divided into the basal body and the export apparatus (Burkinshaw and Strynadka, 2014). The basal body traverses the inner and outer membranes, with the inner membrane ring constituted by PscJ and PscD, while the outer membrane ring is formed by PscC (Burkinshaw and Strynadka, 2014; Notti and Stebbins, 2016). Situated at the basal body lies the export apparatus, assembled from PscR, PscS, PscT, PscU, and PcrD (Deng W. et al., 2017; Notti and Stebbins, 2016). Among these, PscR, PscS, and PscT serve as inner membrane proteins, whereas PscU regulates the secretion switch (Deng W. et al., 2017; Zarivach et al., 2008). PcrD assembles into a ring-like structure that connects the ATPase complex to the secretion pore (Deng W. et al., 2017; Zarivach et al., 2008). The cytoplasmic components encompass the C-ring, composed of PscQ, and the ATPase complex, which includes PscN, PscL, PscO, and PscK. These elements collectively facilitate substrate recognition and secretion (Deng W. et al., 2017; Halder et al., 2019). Notably, PscN assembles into a hexameric structure that interacts with PcrD via the bridging protein PscO (Deng W. et al., 2017; Halder et al., 2019). The inner rod is connected to the inner membrane ring through a “socket” structure, supporting the needle-like protrusion extending from the bacterial surface, with its length regulated by PscP (Deng et al., 2005; Deng W. et al., 2017; Journet et al., 2003). The translocation apparatus is capped at the needle tip by the PcrV tip complex, which, in conjunction with PopD and PopB, forms a translocation pore within the host cell membrane. This pore establishes a direct conduit between the bacterial cytoplasm and the host cell (Romano et al., 2011). The entire T3SS structure is also referred to as the “injectisome,” which shares structural homology with the flagellar hook-basal body complex and operates synergistically to ensure efficient effector secretion and precise host cell invasion (Kubori et al., 1998; Schraidt and Marlovits, 2011).

Current research indicates that at least 25 genes are involved in the regulation of T3SS, among which four regulatory genes (*exsA*, *exsC*, *exsD*, and *exsE*) are located within the five contiguous operons encoding structural components (Hauser, 2009; Yahr and Wolfgang, 2006). Four canonical effector proteins (ExoS, ExoT, ExoU, and ExoY) have been well characterized (Hauser, 2009). Coexpression of *exoS* and *exoU* rarely occurs in a single strain. Strains secreting ExoS induce delayed apoptotic cell death, whereas ExoU-producing strains cause rapid cell lysis (Anantharajah et al., 2016). Moreover, Burstein et al. identified two novel effector proteins, PemA and PemB, through a combination of bioinformatics and experimental validation (Burstein et al., 2015). Most effector proteins rely on specific chaperones for transport (Galle et al., 2012). Chaperones are small molecules that act as “cytoplasmic bodyguards” or “molecular escorts,” binding specifically to substrates (including translocators and T3SS effectors) targeted for secretion through the needle complex. These chaperones play a crucial role in maintaining protein stability, preventing non-specific interactions or premature aggregation within the bacterial cytoplasm, and facilitating substrate recruitment through interactions with various T3SS structural components (Engel and Balachandran, 2009; Horna and Ruiz, 2021b). Additionally, chaperones may contribute to the regulation of the secretion hierarchy (Horna and Ruiz, 2021b).

### 2.2 The regulation system of T3SS

*P. aeruginosa* employs an intricate signaling network to dynamically regulate T3SS expression in response to both extracellular and intracellular cues. Certain pathways are dedicated exclusively to the regulation of T3SS gene expression, whereas others integrate its expression with that of various virulence factors through global regulatory mechanisms (Horna and Ruiz, 2021b; Yahr and Wolfgang, 2006).

**FIGURE 2:**
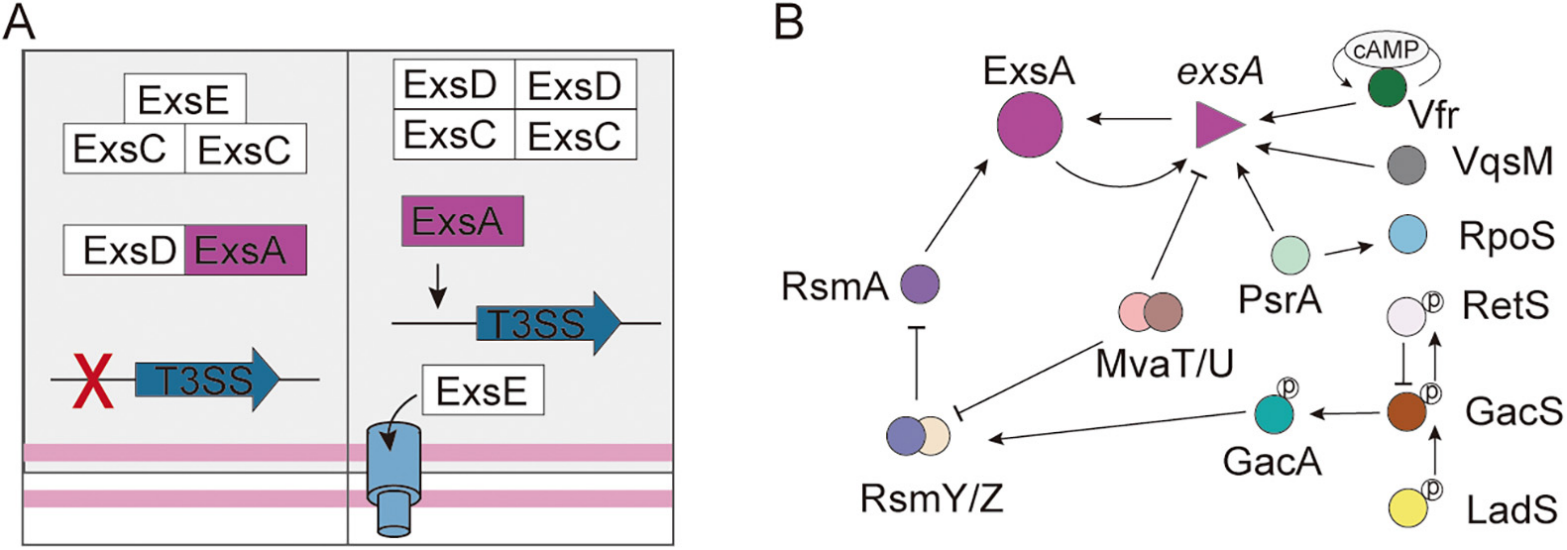
The regulation system of T3SS in *Pseudomonas aeruginosa*. **(A)** The partner-switching mechanism of T3SS controls the DNA-binding activity of ExsA. **(B)** The signaling pathways involved in the regulation of T3SS gene expression. The T-shaped arrowhead indicates inhibition. The solid arrow indicates activation.

Under non-inducing conditions, ExsE interacts with ExsC in a 1:2 stoichiometric ratio, while ExsD forms a 1:1 complex with ExsA, collectively sustaining basal expression levels (Rietsch and Mekalanos, 2006). Under inducing conditions (e.g., Ca^2+^-depleted medium, serum presence, or contact with cells), the negative regulator ExsE is secreted, promoting the formation of a 2:2 ExsC-ExsD complex, which subsequently releases ExsA, thereby enabling the transcriptional activation of operons related to the T3SS (Figure 2A; Rietsch et al., 2005; Thibault et al., 2009; Williams McMackin et al., 2019; Zheng et al., 2007).

The AraC/XylS-family regulator ExsA binds conserved “ExsA box” sequences (AaAAAnwnMygrCynnnmYTGayAk) to activate structural operons, regulatory genes, and effector genes (Burstein et al., 2015; Horna and Ruiz, 2021b; Hovey and Frank, 1995; Yang et al., 2007). ExsA plays a role in the autoregulation of its expression through its binding to the promoter region of the *exsECBA* operon (P_*exsC*_). However, it is important to note that exsA is transcribed from two distinct operons (P*_*exsC*_* and P*_*exsA*_*), with P*_*exsC*_* showing significantly higher transcriptional activity than P*_*exsA*_* (Horna and Ruiz, 2021b; Williams McMackin et al., 2019; Wurtzel et al., 2012). Furthermore, a variety of extrinsic regulatory factors influence the control of T3SS. For instance, PsrA positively regulates the exsCEBA operon and *exoS* (Shen et al., 2006), and directly interacts with the P*_*exsC*_* (Kang et al., 2009). Conversely, PtrA binds to ExsA, thereby repressing the expression of ExsA (Ha et al., 2004). ArtR serves as another repressor of T3SS by reducing exsCEBA transcription (Ha et al., 2004). Several key regulatory systems—including PsrA/RpoS and cAMP/Vfr, the GacSA-RsmYZ-RsmA system, and the regulators VqsM, RetS, LadS, MvaT, and MvaU—coordinate T3SS activity with other virulence factors and resistance mechanisms, forming a sophisticated regulatory framework (Figure 2B; Castang et al., 2008; Deng X. et al., 2017; Ha et al., 2004; Kong et al., 2019). Notably, structural components of the T3SS also play a role in regulation; for instance, the chaperone protein PcrG affects secretion specificity, while the PopN-Pcr1 complex serves as an inhibitor (Lee et al., 2014; Yang et al., 2007). The complex regulatory mechanism allows for precise environmental adaptation.

## 3 Components of the T3SS as therapeutic targets

Given the essential function of T3SS in pathogenesis, there is an increasing agreement that targeting T3SS could revolutionize anti-infective treatments. These strategies specifically disrupt this crucial virulence mechanism, directly reducing bacterial pathogenicity while preserving the balance of microbial ecosystems and lessening the resistance pressures often associated with conventional antibiotics (Anantharajah et al., 2016; Grishin et al., 2018; Horna and Ruiz, 2021a; Pendergrass and May, 2019). This targeted treatment strategy offers a hopeful solution to the increasing problem of infections resistant to multiple drugs.

### 3.1 Effector proteins inhibitors

Some studies on T3SS inhibitors have made significant progress in reducing bacterial virulence by inhibiting the effector proteins. Phillips et al. (2003) were the first to identify phospholipase A2 (PLA2) inhibitors, specifically methyl arachidonyl fluorophosphonate (MAFP) and arachidonyl trifluoromethyl ketone (ATK), which effectively block the enzymatic function of ExoU. Subsequently, pseudolipasin A (Table 1) was identified through high-throughput screening as a potent inhibitor of ExoU. This compound effectively stabilizes the protein in its inactive conformation while exhibiting no cytotoxic effects on the host (Lee et al., 2007). Building upon these findings, optimized arylsulfonamide derivatives showed enhanced targeting efficiency in animal models (Kim et al., 2014; Lee et al., 2007). Furthermore, arylsulfonamide (Table 1) was identified as a promising lead compound that combines the bioactivity of pseudolipasin A with a modular structure conducive to drug optimization (Kim et al., 2014). Overall, these advancements highlight the evolving strategies in creating effective anti-virulence treatments.

**TABLE 1:** The therapeutic targets and inhibitors of T3SS in *Pseudomonas aeruginosa.*

Thera-peutic target	Target	Inhibitors	References
**Effector proteins**			
	ExoU	Pseudolipasin A	Lee et al., 2007
	ExoU	Arylsulfonamide	Kim et al., 2014
	ExoS	Exosin	Arnoldo et al., 2008
	ExoS	STO1101	Pinto et al., 2016
**Regulators**			
	ExsA	*N*-Hydroxybenzimidazoles	Marsden et al., 2016; Grier et al., 2010
	ExsA	Tryptophan derivatives	Shen et al., 2008
	*rsmY/Z*	TS027/TS103	Yamazaki et al., 2012
	ExsA	187R	Fang et al., 2023
	ExsA	II-22	Guan et al., 2024
	ExsA	Alizarin	Kim et al., 2025
**Needle complex components**			
	PscN	Hydroxyquinolines	Anantharajah et al., 2017
	PscC	Thiazolidinones	Anantharajah et al., 2016
	PscF	Phenoxyacetamides	Berube et al., 2017
	PscF	Tanshinones	Feng et al., 2019
	?	Curcumin	Diaz-Guerrero et al., 2025
**Translocation apparatus**			
	PcrV	H1	Sundin et al., 2021

“?” indicates unknown mechanisms.

Using yeast two-hybrid screening, Arnoldo et al. (2008) identified exosin (E2165303) (Table 1) as a specific inhibitor of ExoS. Structural analyses revealed that exosin binds competitively to the active site of the ADP-ribosyltransferase, blocking cytoskeletal disruption in strains expressing ExoS, while having no impact on ExoS-deficient mutants (Saleeb et al., 2018). The optimized derivative exosin-5316 demonstrated five times greater potency and enhanced cellular protection (Arnoldo et al., 2008). Concurrent research also identified STO1101 as a competitor at the active site (Pinto et al., 2016), while structure-activity studies led to the development of improved derivatives (Saleeb et al., 2018).

Future advancements will require a multifaceted approach, including multi-effector combination therapies, structure-guided drug design, and advanced delivery systems (e.g., pulmonary nanoparticles) to enhance efficacy and overcome resistance.

### 3.2 Inhibitors targeting T3SS regulatory system

*N*-Hydroxybenzimidazoles (Table 1) selectively bind to the C-terminal DNA-binding domain of ExsA, inhibiting the activation of T3SS (Marsden et al., 2016; Grier et al., 2010). Shen et al. (2008) reported that tryptophan derivatives (Table 1) (e.g., indole-3-acetic acid) act as effective inhibitors of *exsA* transcription and T3SS expression. Recent studies have demonstrated that the inhibitory effect of alizarin, phenylamino acetamide compound 187R and Thiazole-containing aryl amide compound II-22 on T3SS is mediated through ExsA (Fang et al., 2023; Guan et al., 2024; Kim et al., 2025; Table 1). The phytochemicals TS027 and TS103 (Table 1) modulate the Rsm system by downregulating *rsmY/Z*, which reduces translational repression and facilitates the formation of the RsmA-ExsA complex (Yamazaki et al., 2012). While these findings are promising, they are mainly based on *in vitro* studies, revealing significant gaps in research (Fang et al., 2023; Guan et al., 2024; Kim et al., 2025; Shen et al., 2008; Yamazaki et al., 2012). Future work may combine structural biology and artificial intelligence for optimizing inhibitors, establish dynamic infection models to study host-pathogen interactions, and develop multi-omics-guided intervention strategies targeting epigenetic, signaling, and quorum-sensing pathways for comprehensive regulation (Horna and Ruiz, 2021a; Shreya et al., 2025).

### 3.3 Inhibitors targeting needle complex components

Significant progress has been achieved in targeting the structural components of the T3SS in *P. aeruginosa*. The hydroxyquinoline compound INP1750 (Table 1) has been demonstrated to selectively inhibit the T3SS injectisome, reducing the cytotoxic effects of *P. aeruginosa in vitro* (Anantharajah et al., 2017; Journet et al., 2003). Additionally, thiazolidinones (Table 1) target PscC, an essential structural protein for T3SS basal body assembly (Anantharajah et al., 2016; Notti and Stebbins, 2016). Berube et al. (2017) found that phenoxyacetamides (Table 1) (specifically MBX 1641 and MBX 2359) act as transcriptional inhibitors of *pscF*, significantly decreasing abscess formation. Furthermore, tanshinones (Table 1) competitively bind PscF, disrupting needle assembly without inducing resistance (Feng et al., 2019). Moreover, some studies have demonstrated that curcumin (Table 1) can disrupt the assembly or functionality of the T3SS (Diaz-Guerrero et al., 2025).

### 3.4 Inhibitors targeting translocation apparatus

Sundin et al. (2021) employed a combination of molecular docking and surface plasmon resonance techniques to identify 53 compounds that target PcrV, which resulted in a greater than 60% reduction in bacterial infectivity by obstructing effector translocation (Sato et al., 2011). Among these compounds, the most promising candidate (H1) was selected for the synthesis of analogues (Table 1) and further mechanistic studies.

Although the T3SS is widely acknowledged as a promising target for treating *P. aeruginosa* infections, applying this knowledge in clinical settings remains challenging, as most research concentrating on understanding its mechanisms. Targeting the T3SS of *P. aeruginosa* offers the advantage of avoiding significant selection pressure for antibiotic resistance, though these inhibitors do not directly inhibit bacterial growth. Consequently, there is a pressing need to develop new non-antibacterial treatments specifically for *P. aeruginosa* or to create innovative combination strategies that merge anti-virulence agents with antibacterial medications to effectively tackle this pathogen.

## 4 Innovative T3SS-based vaccine development

The creation of vaccines based on the T3SS offers a preventive strategy that could provide greater benefits compared to treatment methods in some cases. As early as the mid-1950s, scientists discovered LcrV, the needle tip complex protein of Yersinia pestis (Anantharajah et al., 2016), as a protective antigen against plague (Kamei et al., 2011). Studies have shown that deficiency or dysfunction of T3SS in *P. aeruginosa* significantly attenuates virulence, reducing mortality rates from 22% to 4% (Roy-Burman et al., 2001). These findings have resulted in the proposal of vaccine development strategies specifically targeting this molecular machinery. The T3SS has been identified as a promising target for the development of next-generation vaccines, offering potential solutions to the shortcomings of traditional methods.

### 4.1 Live attenuated vaccines based on T3SS

Live attenuated vaccines represent a balanced strategy that maintains immunogenicity while reducing virulence. These formulations, which contain multiple antigens, activate various immune mechanisms in the host and help reduce the selection pressure for antimicrobial-resistant strains (Kamei et al., 2011). However, the process of attenuation might unintentionally remove non-essential antigens, which could lead to immune evasion (Fuentes-Valverde et al., 2022; Santamarina-Fernández et al., 2025). The Killed but metabolically active (KBMA) attenuated strain was developed by deleting the *uvrA* and *uvrB* genes, which encode exonucleases involved in nucleotide excision repair, as well as the T3SS effector genes *exoS* and *exoT* (Meynet et al., 2018; Dubensky et al., 2012; Le Gouëllec et al., 2013). This vaccine introduces a new type of immunogen that uses targeted genotoxic inactivation to stop microbial replication and pathogenicity while maintaining enough metabolic activity to trigger protective immunity (Dubensky et al., 2012). It addresses the drawbacks of traditional live-attenuated vaccines, which have strong immunogenicity but carry a risk of reverting to virulence, and subunit vaccines, which are very safe but less effective (Dubensky et al., 2012). In mouse models, this vaccine produced a wide range of antibodies against OprF and PcrV, and cytokine profiling revealed concurrent Th1/Th2 responses and dominant Th17 activation (Meynet et al., 2018). The vaccine proved to be both safe and effective in models of acute pulmonary infections (Meynet et al., 2018). Future advancements may involve integrating CRISPR-based attenuation for more precise vaccine design (Shi et al., 2024).

### 4.2 Immunization with T3SS components and rational vaccine design

In terms of T3SS component vaccines, progress in structural vaccinology has led to the creation of improved T3SS immunogens. The co-expression of PopB with its chaperone PcrH significantly enhances protein stability and immunogenicity (Schaefers et al., 2018). Intranasal delivery using curdlan adjuvant induces IL-17-mediated protection, while PLGA nanoparticle-encapsulated PopB/PcrV complexes improve antigen presentation and Th17 responses (Schaefers et al., 2018). The L-PaF fusion immunogen demonstrates enhanced protection compared to individual components by combining LTA1 adjuvant with PcrV/PopB antigens (Das et al., 2020; Schaefers et al., 2018). Vaccines targeting PcrV, which neutralizes the secretion apparatus, confer protection against six serotypes, with efficacy improved by 3-oxo-C_12_-HSL (Fakoor et al., 2020b; Golpasha et al., 2015). The PcrV/OprI/Hcp1 and OprF/OprI/PcrV vaccine show enhanced protection in animal models (Fakoor et al., 2020a; Yang et al., 2017). While PscC and PscF represent promising candidate antigens, their optimal adjuvant combinations need further systematic investigation (Goldberg et al., 2022). Emerging technologies, including single-cell omics, will aid in elucidating mucosal memory mechanisms, guiding the development of next-generation “smart vaccines” (Nguyen et al., 2025).

DNA and mRNA vaccines represent promising approaches capable of eliciting robust humoral and cellular immune responses while maintaining favorable safety profiles (Konopka et al., 2025). A quadrivalent DNA vaccine (OprF/OprI/PcrV/PilA) has shown significant efficacy in pneumonia models, inducing Th1-polarized responses with increased levels of IFN-γ/MIP-2 and macrophage recruitment (Saha et al., 2006). Innovative delivery methods using pH-responsive di-aldehyde (PSIH/PEG DA) hydrogel for controlled antigen release have been developed for a bivalent DNA vaccine incorporating OprF epitopes and PcrV antigen (Zhang et al., 2024). This formulation achieves an 83% survival rate while stimulating robust antigen-specific IgG production and enhancing multiple cytokine responses (Zhang et al., 2024). The mRNA vaccines are particularly promising, with constructs encoding PcrV-OprF-I fusion protein showing superior protective efficacy in both burn injury and sepsis models (Zhang et al., 2024). These nucleic acid vaccines offer unique advantages, such as rapid development timelines, modular antigen design, and the ability to elicit comprehensive immune responses without the safety concerns associated with live-attenuated vaccines.

Although several T3SS-based vaccine candidates have been developed, their clinical applications have been hindered by unresolved safety issues and a lack of sufficient evidence for their effectiveness against chronic pulmonary infections. To date, no *P. aeruginosa* vaccine has been approved by regulatory authorities. Nevertheless, foundational research has provided important insights into the mechanisms involved, highlighting the necessity of a thorough understanding of host-pathogen immune interactions for creating vaccines that can generate balanced humoral and cellular immune responses.

## 5 T3SS-based protein delivery system (T3PDS)

**FIGURE 3:**
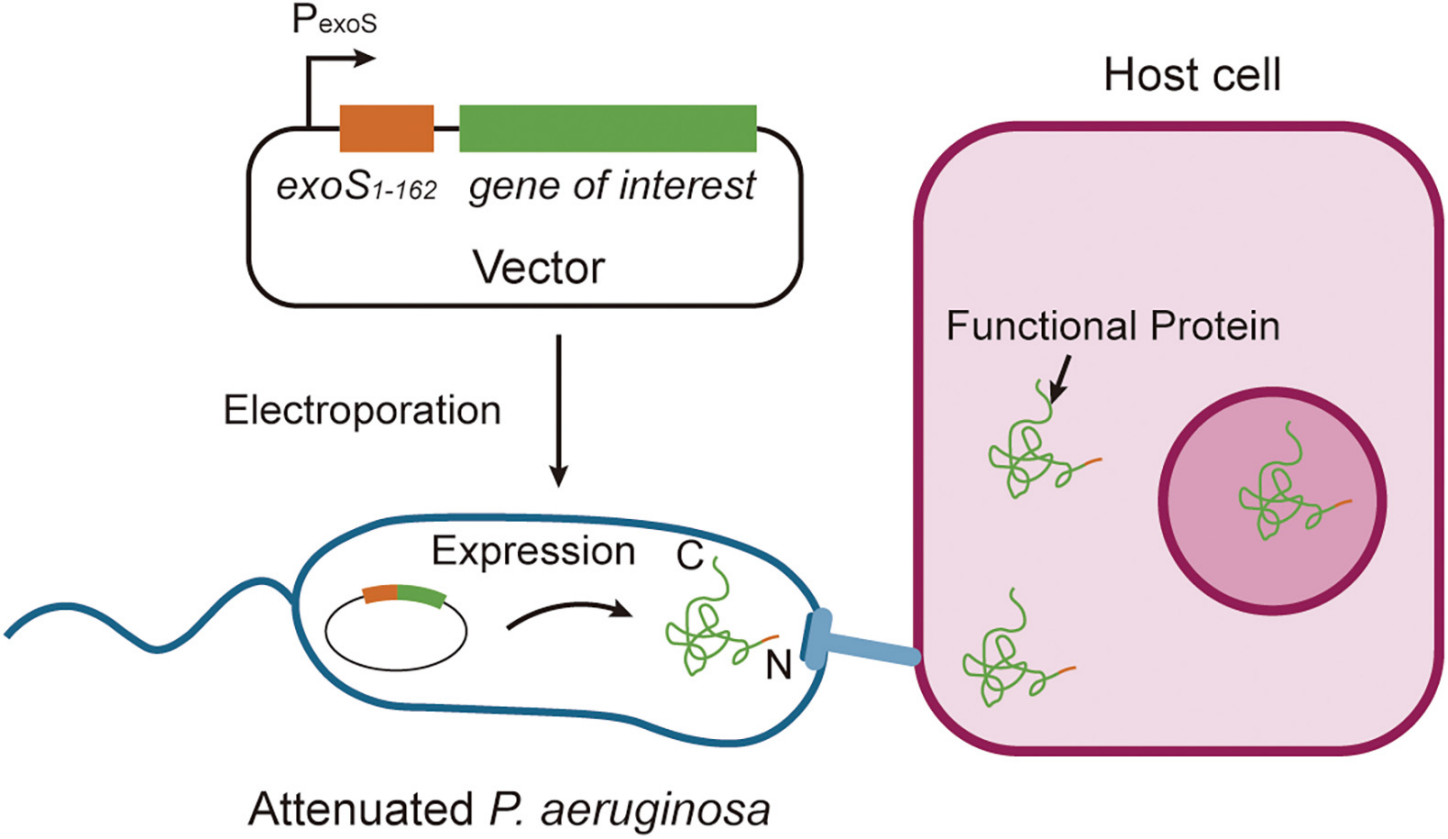
Strategies of functional protein secretion. Proteins of interest are fused to the ExoS_1–54_ secretion signal sequence and cloned into an *Escherichia-Pseudomonas* shuttle expression vector, which is subsequently electroporated into attenuated *Pseudomonas aeruginosa*. Upon bacterial contact with host cells, the chimeric proteins are efficiently injected into mammalian cells through the Type III secretion system, with secretion mediated by the ExoS_1–54_ signal sequence. This system enables precise delivery of recombinant proteins into eukaryotic cells through bacterial infection.

The T3SS is a needle-like macromolecular complex found on the surface of bacteria, which functions to transport four well-known exotoxins: ExoS, ExoT, ExoY, and ExoU (Hauser, 2009). The N-terminal 54-amino acid sequence (ExoS_1–54_) of ExoS serves as a secretion signal peptide, aiding the transmembrane transport of heterologous proteins and enabling the effective delivery of recombinant fusion proteins into the cytoplasm of host cells (Bichsel et al., 2011; Epaulard et al., 2006; Figure 3). This feature makes the T3SS a promising tool for targeted protein delivery.

### 5.1 Vaccine inoculation

The T3PDS represents an innovative antigen delivery system for vaccine inoculation. Its unique mechanism of transmembrane delivery avoids endosomal degradation by directly injecting effector proteins into the host cytoplasm through the needle complex, which enhances MHC class I antigen presentation and activates CD8^+^ T cells (Panthel et al., 2006). The T3SS is exclusively activated upon direct contact between bacteria and host cells (such as antigen-presenting cells), with antigens directionally secreted only at the bacterial pole contacting host cells, thereby preventing inefficient release. Furthermore, metabolic control strategies - such as the application of D-glutamate auxotrophic (ΔmurI) strains - enable bacterial clearance within 10 h post-protein delivery, achieving temporal control (Bai et al., 2018; Cabral et al., 2017). T3PDS offers precise control over the timing and location of antigen expression, significantly boosting immunogenicity (Bai et al., 2018).

Preclinical studies have shown strong efficacy in cancer immunotherapy, with recombinant antigens like TRP-2 or gp100 greatly improving the activation of cytotoxic T lymphocytes (CTLs) and increasing T cell receptor (TCR) diversity (Derouazi et al., 2010). T3PDS in engineered attenuated strains that deliver the OVA_257–264_ epitope effectively present antigens in lymphoid tissues, triggering robust antigen-specific CD8^+^ T cell responses that inhibit tumor growth in B16-OVA melanoma models (Le Gouëllec et al., 2013; Wang et al., 2012). Vaccination leads to the formation of long-lasting effector memory T cell populations, ensuring prolonged immunoprotection (Chauchet et al., 2016). In the context of infectious diseases, T3SS-mediated delivery of the SARS-CoV-2 receptor-binding domain (RBD) protein induces strong humoral immunity in mouse models, with serum IgG/IgM levels exceeding those achieved with standard adjuvants by over three times. Immune sera show enhanced neutralization against pseudotyped viruses and variants of concern (Zhou et al., 2023). The T3PDS offers several key benefits, including precise antigen delivery, reduced cytotoxicity through temporary protein expression, and enhanced biosafety with attenuated bacterial vectors. Collectively, these features push vaccine development toward precision engineering and multifunctional applications, tackling cancer and emerging pathogens.

### 5.2 Gene editing

Gene-editing technologies such as TALENs and CRISPR are typically delivered as DNA or mRNA, which may show variable persistence in target cells. The T3PDS enables transient and dose-dependent modulation of protein activity, providing a temporary yet effective alternative to viral and plasmid vector-based methods. This characteristic is essential for ensuring safety in clinical applications. The T3PDS marks a significant leap in gene editing, achieving approximately 100% (including Cre recombinase and TALENs) within 3 h at a multiplicity of infection (MOI) of 100, while maintaining over 90% efficiency in cells resistant to transfection (Bai et al., 2018; Bichsel et al., 2011). Cell cycle synchronization can enhance editing efficiency to 75%. The main advantages of this technology include overcoming delivery challenges associated with large molecular weight proteins and the elimination of off-target risks linked to sustained nucleic acid expression. Notably, it achieves 2–3 times higher editing efficiency compared to conventional methods in both murine and human stem cells (Jia et al., 2015).

Leveraging biosafety of attenuated strains and modular vector design, the T3PDS has been successfully used in various disease models, including cancer immunotherapy (e.g., delivering PD-L1 blockers), stem cell reprogramming (activating pluripotency genes) and precise editing for monogenic disorders (Bai et al., 2018; Jia et al., 2015; Neeld et al., 2014). Future integration with novel editing tools such as CRISPR-Cas9 and optimization of effector protein targeting strategies may enable tissue-specific gene regulation, providing groundbreaking solutions for genetic diseases and cancer treatment.

### 5.3 Cell fate determination

Cell fate programming refers to the direct alteration of a cell’s gene expression program through external interventions (e.g., delivery of transcription factors), enabling cellular reprogramming or directed differentiation. The T3PDS also has facilitated significant progress in cellular reprogramming and differentiation. Some studies have shown effective delivery of multiple transcription factors, with MyoD promoting myogenic differentiation in about 30% of mouse embryonic fibroblasts (Bichsel et al., 2013), and pluripotency factors (Oct4/Sox2/Nanog) activating pluripotency networks in human fibroblasts and CD34 + hematopoietic stem cells (Berthoin et al., 2016). In cardiac differentiation, the coordinated delivery of GATA4/MEF2c/TBX5 improves cardiomyogenic efficiency in mouse embryonic stem cells, exhibiting synergy with activin A (Bai et al., 2015). While challenges remain regarding subtype heterogeneity and electrophysiological maturity in induced pluripotent stem cell (iPSC)-derived cardiomyocytes, the T3SS allows for the delivery of the generation of specific subtypes (ventricular/pacemaker cells) through single-step delivery, thereby mitigating the risks associated with multi-step genetic manipulation (Jin et al., 2018). Moreover, the T3PDS provides cytotoxic proteins (such as bacterial toxins) and allows for real-time observation of signaling events (including bacterial toxins and factors that induce apoptosis) (Ittig et al., 2015; Wölke et al., 2011). This capability supports innovative research into post-translational modifications and temporary biological processes (Bai et al., 2018; Ittig et al., 2015; Olsen and Mann, 2013). The effective delivery of Rho GTPase effectors and tBID has underscored their significant regulatory functions in influencing cell fate (Bai et al., 2018; Wölke et al., 2011), overcoming the constraints of traditional transfection methods.

When used as a system for protein delivery, the T3SS encounters additional limitations. The remaining bacterial cytotoxicity restricts the extended co-culture of delivery strains with target cells, limiting T3SS-mediated protein delivery to about 5 h (Bai et al., 2018). Additionally, pathogen-associated molecular patterns, which are vital for bacterial survival and cannot be entirely removed, inevitably provoke host inflammatory responses (Tang et al., 2012). These biological constraints currently impede the *in vivo* use of T3SS-based protein delivery systems. Future research will concentrate on two main areas: (1) further reducing the cytotoxicity of *P. aeruginosa* to create safer platforms for T3SS applications, and (2) developing T3PDS that can be controlled in real-time, such as those regulated by light or temperature, to allow for precise timing in delivering proteins to host cells.

## 6 Conclusion

**FIGURE 4:**
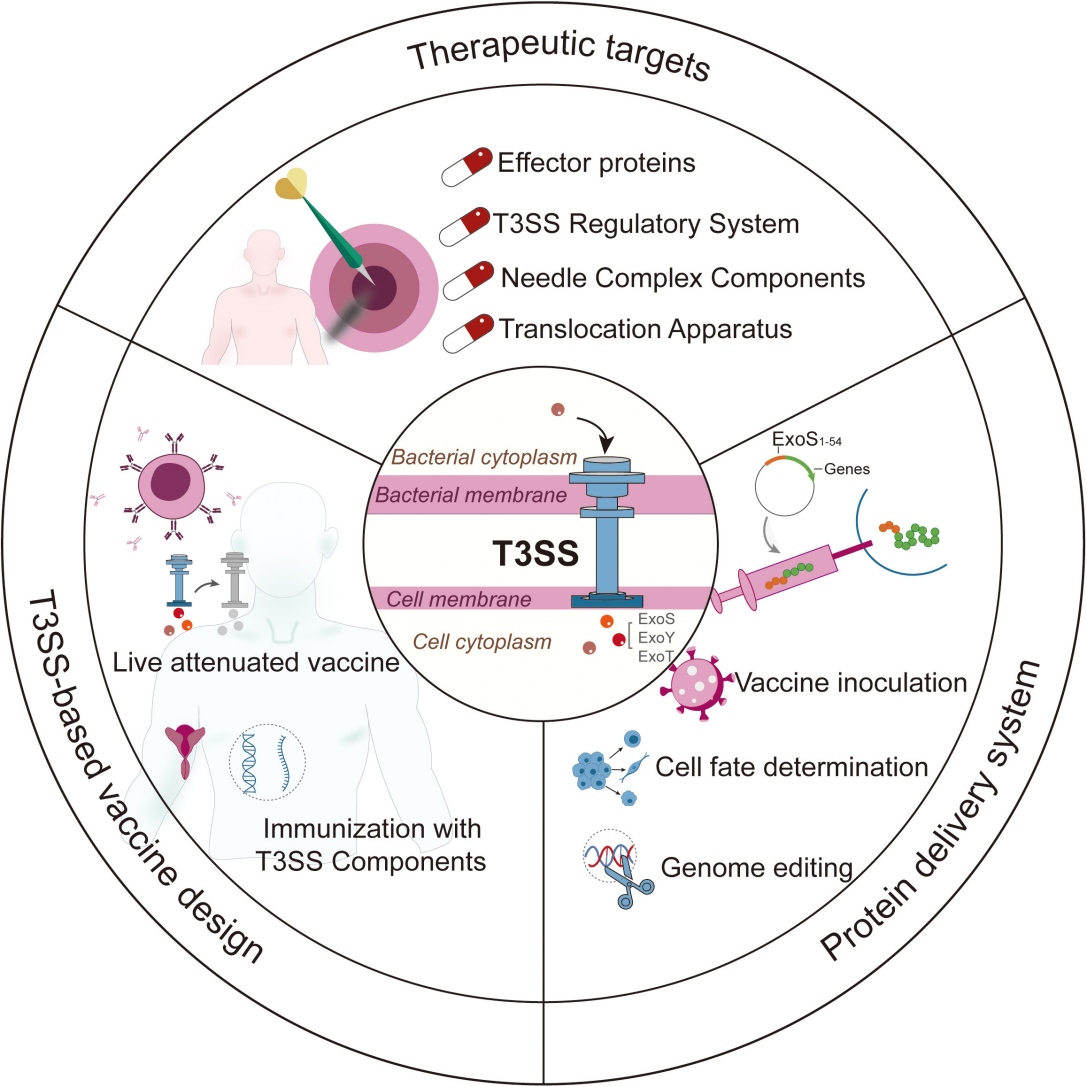
Summary of the applications of T3SS in *Pseudomonas aeruginosa*.

Comprehensive investigations into the structural and functional characteristics of T3SS of *P. aeruginosa* have led to its broad applications in the field of biotechnology (Figure 4). The effector proteins (like ExoS and ExoU) and components of the needle complex are potential therapeutic targets for treating *P. aeruginosa* infections. The system has been adapted for use as a protein delivery platform by utilizing ExoS secretion signals, which allow for applications in vaccine inoculation, gene editing, and cell reprogramming with high precision. While T3SS-based vaccines show potential, they encounter challenges regarding delivery efficiency and safety assessments. Future research should focus on the interactions between host and pathogen to create better antimicrobials and multivalent vaccines, addressing both infectious diseases and regenerative medicine requirements. Furthermore, it is essential to provide biomedical researchers with efficient, transgene-free, and user-friendly protein delivery systems.
